# Thermally Driven Field Emission from Zinc Oxide Wires on a Nanomembrane Used as a Detector for Time-of-Flight Mass Spectrometry

**DOI:** 10.1021/acsomega.3c08932

**Published:** 2024-02-24

**Authors:** Stefanie Haugg, Sylvester Makumi, Sven Velten, Robert Zierold, Zlatan Aksamija, Robert H. Blick

**Affiliations:** † Center for Hybrid Nanostructures (CHyN), Universität Hamburg, 22761 Hamburg, Germany; ‡ Materials Science and Engineering Department, University of Utah, Salt Lake City, 84112 Utah, United States; § Deutsches Elektronen-Synchrotron DESY, 22607 Hamburg, Germany; ∥ The Hamburg Centre for Ultrafast Imaging CUI, 22761 Hamburg, Germany; ⊥ Materials Science and Engineering, College of Engineering, University of Wisconsin–Madison, Madison, 53706 Wisconsin, United States

## Abstract

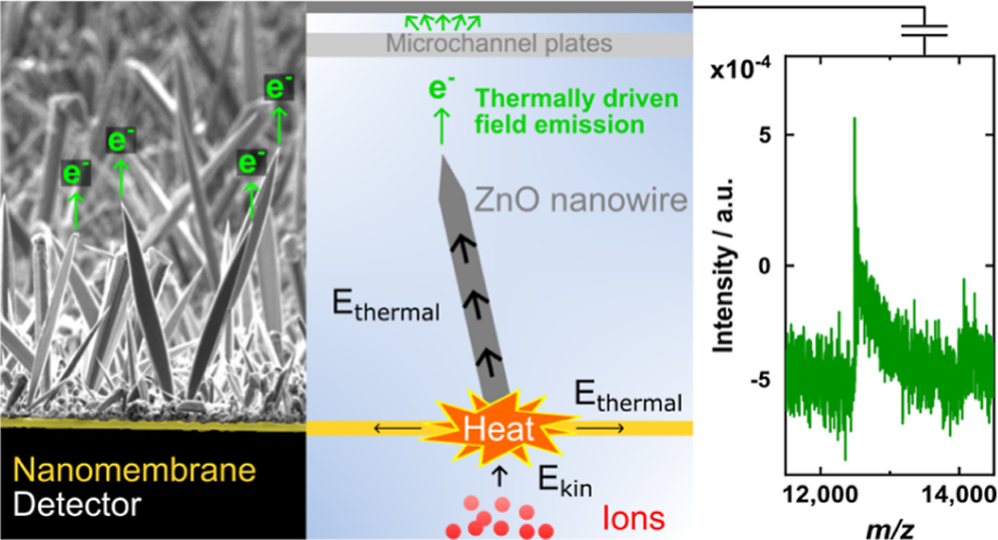

Mass spectrometry is a crucial technology in numerous applications, but it places stringent requirements on the detector to achieve high resolution across a broad spectrum of ion masses. Low-dimensional nanostructures offer opportunities to tailor properties and achieve performance not reachable in bulk materials. Here, an array of sharp zinc oxide wires was directly grown on a 30 nm thin, free-standing silicon nitride nanomembrane to enhance its field emission (FE). The nanomembrane was subsequently used as a matrix-assisted laser desorption/ionization time-of-flight mass spectrometry detector. When ionized biomolecules impinge on the backside of the surface-modified nanomembrane, the current—emitted from the wires on the membrane’s front side—is amplified by the supplied thermal energy, which allows for the detection of the ions. An extensive simulation framework was developed based on a combination of lateral heat diffusion in the nanomembrane, heat diffusion along the wires, and FE, including Schottky barrier lowering, to investigate the impact of wire length and diameter on the FE. Our theoretical model suggests a significant improvement in the overall FE response of the nanomembrane by growing wires on top. Specifically, long thin wires are ideal to enhance the magnitude of the FE signal and to shorten its duration for the fastest response simultaneously, which could facilitate the future application of detectors in mass spectrometry with properties improved by low-dimensional nanostructures.

## 1 Introduction

Field emission (FE) describes the release of electrons from condensed matter into vacuum stimulated by a strong applied electric field. With increasing electric field, the vacuum potential barrier becomes narrow enough for the electrons to escape from the material into vacuum by quantum mechanical tunneling.^1^ In contrast to purely thermionic emission, no external heating source is required, which makes the FE process more energy efficient and facilitates downscaling the size of the field emitter’s application.^2,3^ Typically, FE cathodes are capable of producing emission current densities that are larger than the values achieved by purely thermionic emitters. However, the performance reported for field emitters is known to vary in a wide range and to be highly dependent on the emitting area.^4^ For instance, large and stable FE current densities of over 1400 mA/cm^2^ were achieved at practical applied electric fields below 10 V/μm from carbon nanotubes having a small cathode area of only 0.08 mm^2^.^5^ Moreover, FE can be initialized nearly instantaneously because of the quantum mechanical nature of this emission process, which facilitates fast response times on the order of attoseconds.^6,7^ These outstanding characteristics make FE electron sources exciting candidates for a number of specialized applications, such as for FE displays,^8^ portable X-ray devices,^9,10^ microcomputed tomography scanners,^11^ energy-efficient low-weight space propulsion systems,^12,13^ and as electron beam sources for high-resolution electron microscopy.^7,14^ Another example is the use of field emitter arrays as displacement sensors, such as in accelerometers^15^ and pressure^16−18^ or tactile sensors^19^ because of the fast response times and the exponential dependence of the FE current on the electric field applied to the emitter.

The study presented herein investigates a modification of the FE-based nanomembrane (NM) detector, which was developed for the detection of high-mass protein ions in matrix-assisted laser desorption/ionization time-of-flight (MALDI-TOF) mass spectrometry.^20−25^ Conventional detectors used in MALDI-TOF mass spectrometers are discrete dynodes or microchannel plates (MCPs), which rely on signal generation and subsequent amplification by secondary electron generation. These detectors provide fast response times, high sensitivity, and a sufficiently large sensing area, making them ideal candidates as detectors for MALDI-TOF mass spectrometry.^26,27^ However, the detection efficiency of this detector type is known to decrease considerably with increasing ion mass, which strongly limits the accessible mass range of these devices.^27,28^ It should be noted that the herein investigated NM detector converts the kinetic energy of the ions into thermal energy when hitting the NM’s backside, which can cause mechanical vibrations of the NM and, consequently, a modulated phonon-assisted FE of electrons from the NM’s front side. These FE current fluctuations are subsequently amplified by MCPs in series and allow for the detection of impinging high-mass ions.^20,22^

We tested the performance of a surface-modified NM detector, which was composed of a zinc oxide (ZnO) wire array on top of a 30 nm thin, free-standing silicon nitride (SiN) NM, for MALDI-TOF mass spectrometry measurements of cytochrome *c* [mass-to-charge ratio (*m*/*z*) at about 12,384]. ZnO wires are well-known for their good FE performance, primarily caused by their high aspect ratio that typically generates a large field enhancement factor. Additionally, their good thermal and chemical stability allows for the use of ZnO wires under harsh operating conditions as such as in FE applications.^29,30^ In this study, the ZnO wires were synthesized by catalyst-assisted metal–organic chemical vapor deposition (MOCVD), which allows for their growth at relatively mild growth temperatures (580 °C) and avoids the use of wet chemical processes as needed for solution-based growth methods, having a high potential for destroying the thin, free-standing NM during preparation.^31^ Subsequently, two protein concentrations were tested with the surface-modified NM detector in a MALDI-TOF mass spectrometer. While the applied growth process is compatible with the NM substrate, we found that the control over the ZnO wire dimensions and their distribution is limited. Therefore, we used the mean wire dimensions as the starting point for extensive simulations to examine the impact of the ZnO wires on the performance of the NM detector. The theoretical calculations reveal potential ways to further improve the experimental performance of the surface-modified NM detector in the future by investigating other growth processes that could allow for the precise tailoring of the ZnO field emitter dimensions.

## 2 Materials and Methods

### 2.1 Sample Preparation

The ZnO wires were directly grown on a 30 nm thin SiN NM (3 × 3 mm^2^, from Silson) by MOCVD. For catalyst deposition, the freely suspended SiN NM was sputtered with gold for 60 s through a mesh-like shadow mask, which yielded a mean film thickness of about 6 nm. Subsequently, the MOCVD of ZnO wires was carried out in a horizontal three-zone tube furnace (OTF-1200X-III-UL, MTI corporation) using a precursor combination of 2.4 g zinc acetylacetonate hydrate (Sigma-Aldrich) and 83 sccm oxygen, with 100 sccm argon as the transport gas. A growth temperature of 580 °C was used for the growth duration of 12 h. Further details on the used ZnO wire growth process can be found in an earlier publication.^31^

### 2.2 NM Detector Setup

The NM detector measured ionized proteins in a modified MALDI-TOF mass spectrometer (Voyager-DE STR, Applied Biosystems). For protein preparation, the standard protein cytochrome *c* from the equine heart (molecular weight 12,384 u) was dissolved in 0.1% trifluoroacetic acid in water. The concentration of the protein solution was adjusted to 16 and 153 μmol/L, which are referred to as low and high concentration, respectively. For the MALDI matrix preparation, a modified version of the method by Signor and Erba was used, which combines the thin layer technique generated from α-cyano-4-hydroxycinnamic acid (α-CHCA) with a mixture of the two MALDI matrix substances α-CHCA and 2,5-dihydroxybenzoic acid (DHB, both from Bruker Daltonics).^32^ A detailed description of the used MALDI matrix preparation protocol is given elsewhere.^33^ The protein–matrix mixture was transferred to the MALDI target plate by manual pipetting. After the MALDI samples were dried in ambient conditions, the target plate was transferred into the high-vacuum ion source chamber (<2 × 10^–5^ Pa) of the MALDI-TOF instrument.

The mass spectrometry measurements were carried out in linear positive ion mode using an acceleration voltage of *U* = 25 kV, a delayed ion extraction time of 485 ns, and a laser firing rate of 20 Hz. A load-lock chamber was installed to allow for convenient detector exchange without venting the complete MALDI-TOF instrument. A conventional MCP detector—consisting of two MCPs assembled in Chevron configuration (Hamamatsu Photonics)—was used. The measured flight time (*t*) of the ions was converted to the corresponding *m*/*z* value using the following equation
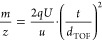
with the electron charge *q* = 1.602 × 10^–19^ C, the atomic mass unit *u* = 1.661 × 10^–27^ kg, and the adjusted flight path length *d*_TOF_ = 2.252 m.^34^ The *m*/*z* values of the peak locations in each mass spectrum were extracted using the “findpeaks” function from MATLAB (MathWorks). Subsequently, all the determined *m*/*z* values were plotted in a histogram for each protein concentration. The herein presented individual mass spectra were additionally treated with the MATLAB function “msbackadj” for baseline correction and with “mslowess” for signal smoothing.

### 2.3 Simulation Methods

Since the NMs used as detectors are thin (thickness below 100 nm) with lateral dimensions far exceeding the thickness, we modeled them by solving a two-dimensional heat diffusion equation in the (*x*, *y*) plane of the NM
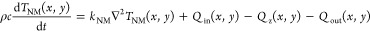
where ρ*c* is the volumetric heat capacity, given by the product of density and specific heat, while *k*_NM_ is the thermal conductivity of the NM. *Q*_in_(*x*,*y*) is the heat converted from kinetic energy of impinging proteins on the back of the NM, while *Q*_Z_(*x*,*y*) is the heat flowing up into the ZnO wires, and *Q*_out_(*x*,*y*) is the heat loss due to radiation from both top and bottom of the NM, computed using the Stefan–Boltzmann law while assuming the environment is at room temperature. We used finite differences to approximate the Laplacian operator and an explicit Runge–Kutta method for the time-dependence.

For heat flux along the ZnO wires, we solved the Fourier Law heat conduction equation in the *z* direction iteratively at each time step, using the solution at the bottom of the wire obtained from the temperature of the NM at the center of the wire. Assuming constant heat flux in the vertical direction along the wire, we have
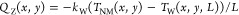
where *k*_W_ represents the thickness-dependent thermal conductivity of the wires. The FE current from the tips of the wires is a function of the tip temperature *T*_W_(*L*), where *L* is the length of the wire.

The FE current is computed by combining Fowler–Nordheim tunneling, thermionic emission, and Schottky barrier lowering, as described in detail in our previous work.^22,25^ The total heat flux out due to FE is given by *Q*_W_(*x*,*y*) = *E*_avg_/*qJ*(*T*_W_(*x*,*y*,*L*)), where *E*_avg_ is the average energy of emitted electrons. At every time step, the two equations for *Q*_Z_(*x*,*y*) from the NM into the wires and *Q*_W_(*x*,*y*) for flux out of the wires at every point on the NM are iterated, varying the temperature at the tips of wires until self-consistency between heat flux from NM and loss from wire tips due to FE is achieved.

## 3 Results and Discussion

### 3.1 NM Detector Measurement

**Figure 1:**
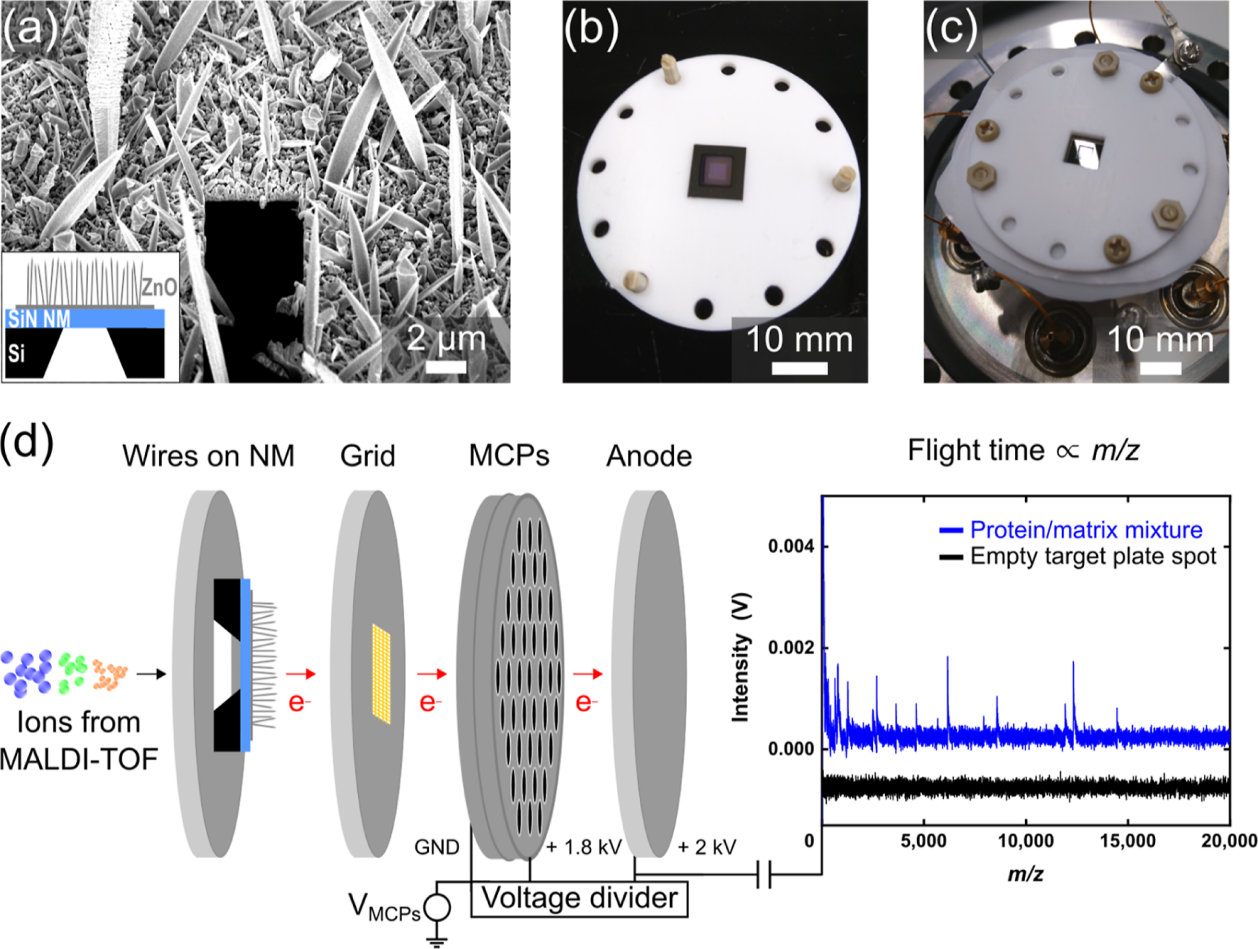
Experimental setup of the NM detector measurement. (a) ZnO wires on the SiN NM are shown in the SEM image, and the sample’s structure is schematically presented in the inset. Photographs of the (b) front and (c) back side of the NM sample being still intact after the mass spectrometry measurement. (d) The main components of the NM detector setup are displayed schematically: NM, grid, MCPs, and the anode. A mass spectrum can be measured when ions hit the backside of the NM (plotted in blue). In contrast, no significant signal change is observed without ions bombarding the NM (black). Note that no voltage was applied to the NM and the grid.

The morphology of the randomly distributed, sharp ZnO wires on a SiN NM and the schematic structure of the sample are displayed in Figure 1a. The mean ZnO wire length and diameter of about 50 and 2.3 μm, respectively, were extracted in a previous study for the herein used growth process.^31^ Note that the wire dimensions vary over a relative broad range of values, which can be seen in the histograms in Figure S1 of the Supporting Information. The X-ray diffraction (XRD) analysis of a ZnO wire array, which was grown on a SiN bulk substrate under the same process conditions, showed peaks that correspond to the hexagonal structure of ZnO. Details on the XRD measurement are presented in Figure S2 of the Supporting Information. Photographs of the top and of the bottom side of the NM detector after the mass spectrometry measurements are displayed in Figure 1b,c, respectively, demonstrating that the NM remained intact. The structure of the NM detector is schematically shown in Figure 1d and consists of the following main components: ZnO wires on a SiN NM separated from a grid by a 250 μm thick poly(tetrafluoroethylene) sheet, two MCPs arranged in Chevron configuration, and a metal anode. Note that the described NM detector assembly is based on previous publications.^20,22,23^

One high-voltage power supply applied 1800 V to the MCP output and 2000 V to the anode through a voltage divider circuit, while the MCP input was connected to the ground. No voltage was applied to the NM and to the grid electrode. Before the voltage was supplied to the MCPs for the initial measurement, the NM detector was kept in the high-vacuum chamber of the MALDI-TOF instrument (<2 × 10^–5^ Pa) for several days to allow for proper degassing, which reduces the risk of electrical discharge between the electrodes of the detector assembly.^35^ An external oscilloscope was used to measure the signal amplitude as a function of the flight time, with the oscilloscope’s input set to 1 MΩ for impedance matching. The capacitor between the anode and the external oscilloscope suppressed any DC current noise to acquire the AC signal generated by the changes in the electron signal emitted from the ZnO wires caused by ion impacts on the NM’s backside.^20^ The trigger signal from the mass spectrometer was recorded to mark the zero-flight time, and the electron current fluctuations measured at the anode were acquired for 200 μs using a rate of 500 × 10^6^ samples/s. A single mass spectrum was obtained by averaging the signal generated by 100 laser shots, and 100 individual mass spectra were measured for both the low and high protein concentrations. For comparison, a mass spectrum measured at an empty target spot (plotted in black) and one that was acquired for the protein–matrix mixture (low concentration, blue) are presented in Figure 1d. Note, the measurement of the empty target spot was shifted by −0.001 V for a clear visibility of both spectra. It becomes evident from comparing the two mass spectra that the signal variations only appeared when the ions hit the backside of the NM.

**Figure 2:**
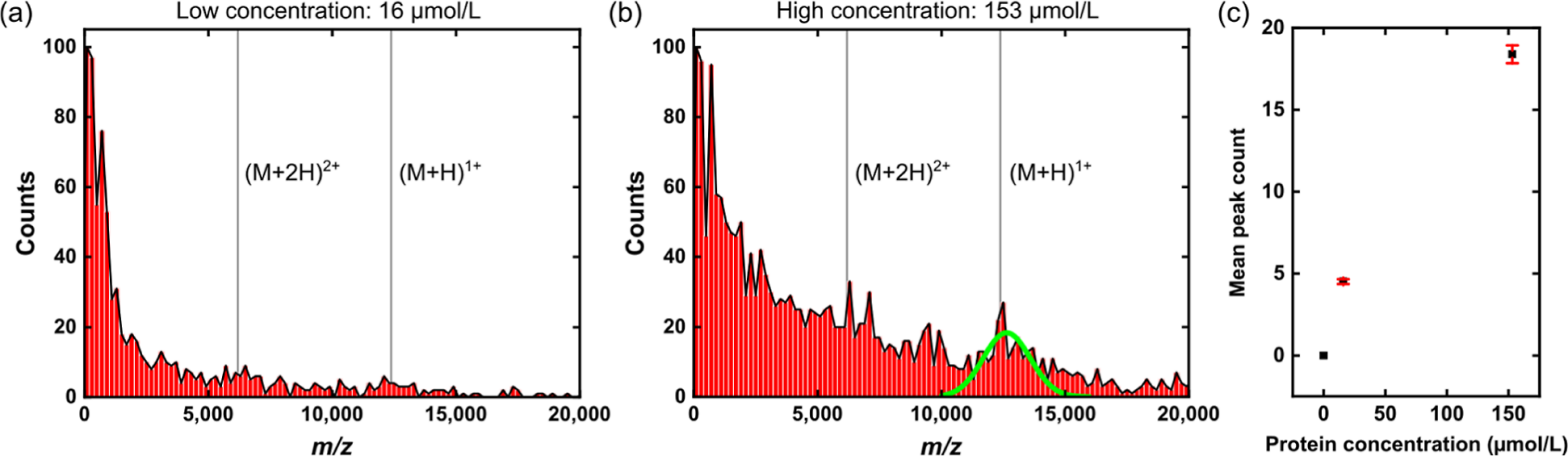
Histograms of the MALDI-TOF mass spectrum for cytochrome *c* measured with the NM detector. The peak values for each histogram were extracted from 100 individual mass spectra. A cytochrome *c* concentration of 16 and 153 μmol/L were used for the measurements that are summarized in the histograms (a,b), respectively. The expected *m*/*z* values of the singly ((M+H)^1+^) and of the doubly charged ions ((M+2H)^2+^) are indicated by the vertical gray lines. (c) Mean peak count increased with the protein concentration.

The surface-modified NM detector was used to measure 100 mass spectra of the protein–matrix mixture for both protein concentrations. First, the *m*/*z* values for the peaks appearing in each mass spectrum were extracted. Subsequently, the *m*/*z* values found for the peaks in each of the 100 mass spectra were plotted in the histograms for low and high cytochrome *c* concentration in Figure 2a,b, respectively. Note, a bin size of *m*/*z* 200 was used. The presentation of the data in the form of histograms was chosen to show that the abundance of the peaks varies with the *m*/*z* value. For comparison, Figure S3 of the Supporting Information displays an overlay of the 100 mass spectra measured for each protein concentration. The black curves represent the counts for each bin center and were overlaid to guide the eye. As expected, the largest number of counts appeared for *m*/*z* values below 1000, which can be assigned to the low-mass matrix ions. The matrix molecules were present in a much higher concentration in the MALDI sample than that in the protein molecules, which is typical for the MALDI method, allowing for the generation of intact protein ions of low charge states.^34^ Consequently, the probability of the matrix ions being sensed with the surface-modified NM detector is particularly high because of their abundance.

The vertical gray lines in Figure 2a,b indicate the *m*/*z* values that are expected to appear for the singly ((M+H)^1+^) and doubly charged cytochrome *c* ((M+2H)^2+^). Evidently, the peak count was enhanced for the measurement of the high protein concentration (Figure 2b) compared to the results for the lower cytochrome *c* concentration (Figure 2a). This observation suggests that the probability for ion detection with the surface-modified NM increases when more protein ions approach the backside of the NM detector. Note that the matrix ion abundance is still much larger than the number of protein ions for the high analyte concentration, reflected in the higher number of counts collected for low *m*/*z* values (<1000). More thermal energy is possibly deposited in the NM when a larger number of ions hit the backside of the NM detector. Subsequently, the signal intensity generated by FE from the ZnO wires on the NM’s front side gets enhanced through the immense amount of thermal energy supplied by the impinging ions.

However, a detectable change in FE current can only be measured if sufficient energy is provided to the NM, which could explain the observed dependence of the detection probability on the protein concentration and indicate a threshold-like sensing behavior of the surface-modified NM detector. Note that the background that appears in both histograms was possibly generated by fragments of the matrix–protein mixture. Moreover, the inhomogeneous length-to-diameter distribution of the randomly arranged wires (Figure 1a) could have caused a location-dependent variation of the threshold for FE across the NM, and hence, of the detector sensitivity.

A Gaussian fit (green line in Figure 2b) was overlaid to extract the mean number of counts observed for the singly charged cytochrome *c* ion (zoomed view in Figure S4 of the Supporting Information). A mean count number of about 18 was observed for a protein concentration of 153 μmol/L (high), in contrast to the mean count number of about 5 for 16 μmol/L (low). Thus, the number of counts was enhanced by a factor of about 4 when the protein concentration increased by a factor of roughly 10. This concentration-dependent change in detection probability is summarized in Figure 2c and presents an initial indication of the sensitivity of the detector. Further increase of the protein concentration could likely lead to a saturation of the peak count, since too high analyte concentrations can suppress the effect of the matrix by hindering the ionization efficiency of the MALDI process.^34,36^ Note that the peak intensity extracted from the mass spectra cannot be used as a measure for the detector’s sensitivity as it does not vary with the protein concentration, which is shown in Figure S5 of the Supporting Information.

### 3.2 Simulation Results

To understand the thermally driven FE effect in a SiN NM and the role of ZnO wires on its surface, we have developed an extensive simulation framework based on a combination of lateral heat diffusion in the NM, heat diffusion along the wires, and FE including the Schottky barrier lowering from the tip of the wires. Parameters, including thickness-dependent thermal conductivity of the NM and wires, are calculated using models we previously documented.^22,25,37^ In the simulation, as described further in the subsection Simulation Methods, heat is supplied to the membrane by converting the kinetic energy of a pulse of impinging molecules. Subsequently, the thermal energy is removed by electrons emitted from the tip of the wires on the other side of the NM. The calculations have been done assuming a doping concentration of 10^16^ cm^–3^. The impact of wire lengths and thickness on FE has been investigated by considering wires of lengths 10, 50, and 100 μm and diameters of 1.3, 2.3, and 3.3 μm.

**Figure 3:**
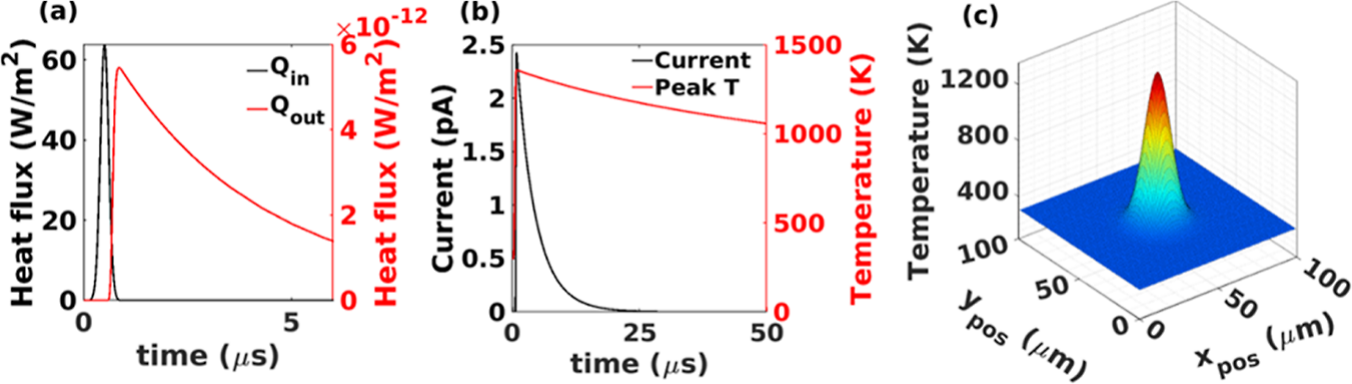
(a) Graph of heat flux density into the NM caused by the kinetic energy of the bombarding protein molecules and the emitted heat flux density as a function of time. (b) Resulting FE current emitted from the NM and the peak temperature of the NM as a function of time. (c) Temperature profile of the NM at 0.9 μs. The temperature is at a maximum of ∼1300 K at the center and reduces to room temperature as we move laterally.

As shown in Figure 3a,b, the heat input, *Q*_in_, that is caused by the impact of proteins on the back of the NM, increases to a peak and then drops to zero after approximately 0.9 μs. This heat flows across the NM to the wires, where it propagates through lattice vibrations (phonons) whose group velocity can be taken to be the speed of sound in the material.^37^ Since the thickness of the NM, taken to be 30 nm in our simulations to match the measurements, is much smaller than the length of the wires, we assume that transport across the NM is much more rapid than the heat diffusion along the wires. In contrast, in-plane transport is governed by two-dimensional heat diffusion with thermal conductivity impacted by phonons scattering from atomically rough top and bottom surfaces. Because of this large anisotropy, the temperature of the NM increases sharply to a maximum, then drops at a lower rate as heat flows laterally out of the NM and perpendicularly along the wires. At the tips of the wires, heat causes an increase in FE. The emitted electrons have energies comparable to the height of the energy barrier at the surface, given by the electron affinity χ_ZnO_ of ZnO, and thus remove considerable energy, with the total heat flux approximated by *Q*_W_ ≈ χ_ZnO_*J*_FE_/*q*. High temperature and sharp wire tips facilitate the emission of electrons by both thermionic emission and Fowler–Nordheim tunneling effect.^25^ The resulting FE current increases to a maximum when the temperature of the wires is maximum and decays exponentially to zero after about 25 μs. FE enhances the cooling of the NM and accelerates the decay in temperature relative to lateral heat diffusion alone, indicated by the sharp temperature peak in Figure 3c. The Figures S6 and S7 in the Supporting Information demonstrate the variation of the Fermi level in dependence on temperature and doping concentration.

**Figure 4:**
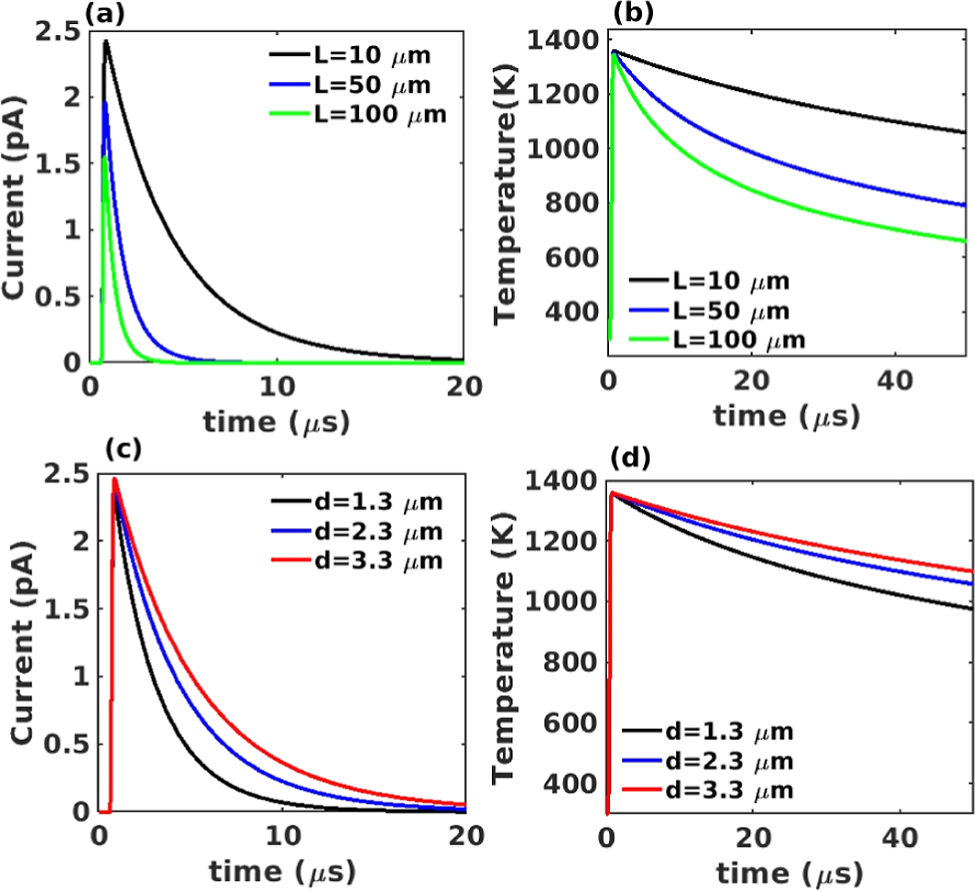
(a) Graph of FE current (*J*_FE_) against time at different wire lengths (*L*). Increasing the length of the wire decreases the peak value of the current but also reduces the time needed for the current to return to zero, which can help to improve selectivity. (b) Graph of temperature at the tips of wires against time at different wire lengths (*L*). Increasing the length of the wire decreases the temperature trend at the tip. (c) Graph of current as a function of time at different wire diameters (*d*). Decreasing the wire’s diameter reduces the decay time of the current. (d) Graph of the temperature of wires against time at different wire diameters (*d*). Decreasing the diameter of the wire lowers the temperature trend at wire tips, reducing the FE current, resulting in accelerated decay and faster response. Note that a uniform wire diameter of 2.3 μm was assumed for the theoretical calculations of (a,b), and the wire length was fixed to 10 μm for (c,d). We also analyzed the impact of having a distribution of wire diameters and lengths instead of uniform values across the entire NM. The results are shown in Figures S8 and S9 of the Supporting Information, and they indicate that the current stays close to the value obtained from wires with uniform diameter and length as long as the distribution is symmetric.

From the simulation results, we observe a decrease in the peak FE current with increasing wire length, as illustrated in Figure 4a. This can be attributed to the temperature drop with increased distance from the NM to the tips of the wires, as shown in Figure 4b. However, increasing the length of wires reduces the time taken for the current to decay to zero (Figure 4a). On the other hand, thinner wires show a higher rate of cooling and a shorter time for the current to drop to zero without affecting the peak current value, as shown in Figure 4c. This can be attributed to thinner wires having lower thermal conductivity, resulting in lower tip temperature, confirmed by Figure 4d, which drives FE down faster after the initial peak. We conclude from the theoretical modeling that the ZnO wires have a strong positive impact on the overall FE response of NMs and that long, thin wires are ideal for simultaneously enhancing the magnitude of the FE signal and shortening its duration for the fastest response.

### 3.3 Comparison of Experiment and Simulation Results

For comparison of the experiment with the simulation results, the rise and fall time of the peak that appeared for the singly charged cytochrome *c* were extracted from the experimental data and summarized in Table S1 of the Supporting Information. Further investigation focused only on the fall time since the steep rise time (<0.1 μs) has no considerable impact on the resolution. A fall time of (1.9 ± 1.2) μs and of (1.3 ± 1.2) μs were found for the low and high protein concentrations, respectively. Thus, the time resolution appears to be independent of the protein concentration as the extracted fall times vary within their standard deviations. These fall times correspond to a Δ*m*/*z* of about (422 ± 266) and (289 ± 266) for low and high protein concentrations, respectively. Subsequently, a rough estimate for the detector’s mass resolution (*m*/Δ*m*) of about 29 and 43 can be derived for the low and high protein concentrations, respectively. Compared to previous studies, which used pristine membranes with a thickness below 200 nm, the resolution of the surface-modified NM detector is lower, as summarized in Figure S10 of the Supporting Information.^22,25^ Note that voltages of over 1 kV had to be applied to the pristine membranes from previous work, but the surface-modified NM in this study was operated bias free. The observed peak shape—namely, a steep increase in intensity, followed by an exponential decrease as predicted by the simulation results in Figure 4—is shown exemplarily in Figure S11 of the Supporting Information. Compared to a conventional MCP detector without a NM and without proprietary software processing, which was used in the same mass spectrometer, a larger fall time of about 5 μs was found, which corresponds to a Δ*m*/*z* of about 1153 (Figure S12 of the Supporting Information).

**Figure 5:**
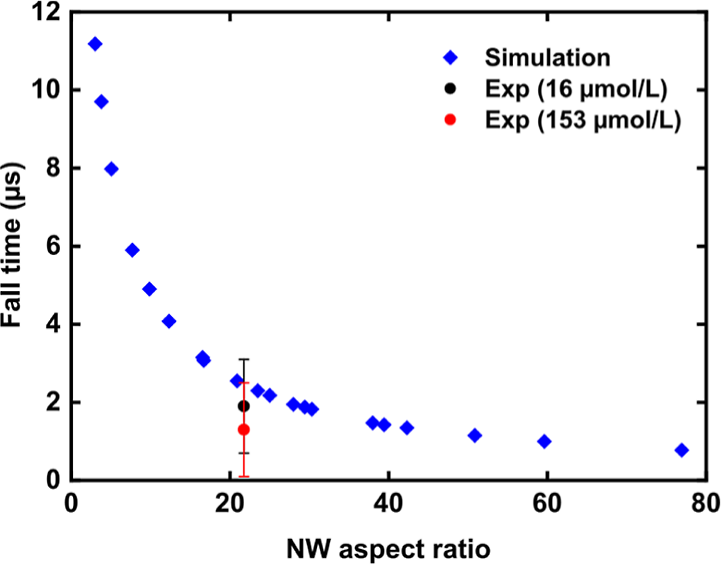
Comparison of the simulation results to the experimental fall times for both protein concentrations in dependence on the wire aspect ratio. The experimental fall times correspond to a Δ*m*/*z* of about (422 ± 266) and (289 ± 266) for low and high protein concentrations, respectively.

As shown in Figure 5, an exponential decrease of the fall time with increasing wire length is expected according to the simulation results (blue diamonds). Compared to the mean experimental fall times for low (black dots) and high protein (red dots) concentration, a slightly longer fall time is predicted by the simulation but in the range of the error. For the aspect ratio of 21.7, which was calculated from the mean length (50 μm) and diameter (2.3 μm) of the measured wire array, a theoretical fall time of 2.5 μs was found. This observation suggests that the wires that determine the FE, and therefore, the detector response, have a larger aspect ratio. Typically, the emitters having the largest geometrical field enhancement factors, which are usually the highest and sharpest wires, determine the FE behavior of an array.^38−40^ Therefore, we conclude that the outlier in geometrical size, such as the ZnO wires shown in Figure S13 of the Supporting Information, are mainly responsible for the FE and hence, for the detector response of the experimentally tested surface-modified NM. Possibly, only a small fraction of the wire array actually contributes to the FE signal because of the broad distribution of wire dimensions, as shown in Figure S1 of the Supporting Information. This effect may explain the lower mass resolution of the surface-modified NM detector compared to that reported in earlier work with uncoated membranes.

The theoretical fall times gradually plateau beyond aspect ratios of 80. There are, furthermore, two limitations on the aspect ratio. On the one hand, if the diameter is reduced below the mean free path of phonons, which is around 100 nm in ZnO,^41^ then boundary scattering reduces the thermal conductivity of the wires, limiting further gains in performance. On the other hand, the experimental wire dimensions are limited by the growth process. The largest aspect ratio we observed was about 64 (Figure S13 of the Supporting Information). Thus, the optimal wire dimensions obtained by the simulations cannot be achieved by the currently used NM-compatible fabrication method. The limited control over the wire dimensions did not allow for a simultaneous increase in wire length and decrease in diameter, as found in a previous study.^31^ For future advancement of the surface-modified NM detector, other growth methods may be explored based on our simulation results.

## 4 Conclusions

We generated a ZnO wire array on a 30 nm thin, free-standing SiN NM, which was subsequently used in a FE-based detector for MALDI-TOF mass spectrometry operated with a bias-free NM. The surface-modified NM detector showed a dependence of the detection probability on the protein concentration, namely, the number of counts collected for the peaks—which were presumably generated by the singly charged cytochrome *c* ion—improved with the number of protein ions. This observation is likely related to the increase in thermal energy deposited in the NM when a larger amount of analyte ions hits the NM’s backside, which is supposed to enhance the FE signal from the ZnO wires. Additionally, an extensive simulation framework was developed that is based on a combination of lateral heat diffusion in the NM, heat diffusion along the wires, and FE, including Schottky barrier lowering from the tips of the wires to theoretically study the impact of wire dimensions on the performance of the surface-modified NM detector. The simulations suggest that the FE from the ZnO wires enhances the NM’s cooling by accelerating the temperature decay relative to lateral heat diffusion alone. Furthermore, the theoretical calculations indicate that the ZnO wires have a strong positive effect on the overall response of the surface-modified NM detector. Note, the simulation results reveal that long and thin wires would be ideal for both, enhancing the magnitude of the FE signal and shortening the duration of the detector response time. Tailoring the ZnO wires according to the dimensions suggested by the simulations could lead to an enhanced signal response from the surface-modified NM detector in the future. Since this is not possible with the herein applied growth method, other fabrication methods for field emitters may be explored, such as the growth of AlGaN nanowires by molecular beam epitaxy^42^ or the template-assisted deposition of ordered carbon nanotube structures,^43^ which could provide improved control over the emitter dimensions. However, the compatibility of such techniques with thin, free-standing NM substrates still has to be tested. Furthermore, operating several surface-modified NMs in parallel would increase the active detection area, which could lead to an enhanced detection probability for the ionized biomolecules. This work paves the way toward mass spectrometry detectors with properties enhanced by low-dimensional nanostructures.

## Supporting Information Available

Additional details on the ZnO wires, mass spectrometry measurements, and simulation results (PDF)
