# Correction to “Thermally Driven Field Emission from ZnO Wires on a Nanomembrane Used as a Detector for Time-of-Flight Mass Spectrometry”

**DOI:** 10.1021/acsomega.4c05525

**Published:** 2024-06-26

**Authors:** Stefanie Haugg, Sylvester Makumi, Sven Velten, Robert Zierold, Zlatan Aksamija, Robert H. Blick

**Affiliations:** † Center for Hybrid Nanostructures (CHyN), Universität Hamburg, 22761 Hamburg, Germany; ‡ Materials Science and Engineering Department, University of Utah, Salt Lake City, Utah 84112, United States; § Deutsches Elektronen-Synchrotron DESY, 22607 Hamburg, Germany; ∥ The Hamburg Centre for Ultrafast Imaging CUI, 22761 Hamburg, Germany; ⊥ Materials Science and Engineering, College of Engineering, University of Wisconsin-Madison, Madison, Wisconsin 53706, United States

We are issuing a correction to the acknowledgment section of the original manuscript (DOI: 10.1021/acsomega.3c08932). We apologize for the oversight.

Original acknowledgment

This work was funded by the Deutsche Forschungsgemeinschaft (DFG, German Research Foundation) – project number 469222030 and by the National Science Foundation CDS&E award number 2302879.

Revised acknowledgment

This work was funded by the Deutsche Forschungsgemeinschaft (DFG, German Research Foundation) – project number 469222030 and by the National Science Foundation under Grant No. DMR-2302879.

