# Supplementary material for: Thermally Driven Field Emission from Zinc Oxide Wires on a Nanomembrane Used as a Detector for Time-of-Flight Mass Spectrometry
Source: ACS Omega. 2024 Feb 24;9(9):10602–9. doi: 10.1021/acsomega.3c08932 (PMC10918783; doi:10.1021/acsomega.3c08932)
Supplement: Supplementary file 1 — ao3c08932_si_001.pdf [file ao3c08932_si_001.pdf]

Supporting Information

Thermally Driven Field Emission from Zinc Oxide Wires on a Nanomembrane Used as a Detector for Time-of-Flight Mass Spectrometry

Stefanie Haugg[1,‡,*], Sylvester Makumi[2,‡], Sven Velten[3,4], Robert Zierold[1], Zlatan Aksamija[2], and Robert H. Blick[1,5]

[1] Center for Hybrid Nanostructures (CHyN), Universität Hamburg, 22761 Hamburg, Germany
[2] Materials Science and Engineering Department, University of Utah, Salt Lake City, Utah 84112, USA
[3] Deutsches Elektronen-Synchrotron DESY, 22607 Hamburg, Germany
[4] The Hamburg Centre for Ultrafast Imaging CUI, 22761 Hamburg, Germany
[5] Materials Science and Engineering, College of Engineering, University of Wisconsin-Madison, Madison, Wisconsin 53706, USA

[*] Corresponding author
[‡] Both authors equally contributed to the work.

Zinc oxide (ZnO) wire dimensions

The dimensions of the ZnO wires were measured in SEM images using ImageJ [1]. The presented values for the tilt angle describe the angular difference to a vertically aligned wire.

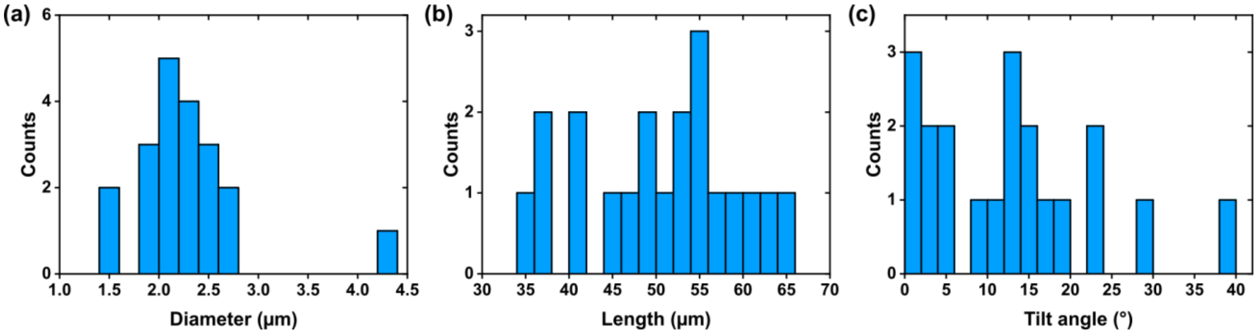

Figure S1. (a) Diameter, (b) length, and (c) tilt angle distribution of the ZnO wires.

X-ray diffraction analysis of ZnO wires

Crystal structure information was obtained by X-ray diffraction (XRD) using a Rigaku SmartLab X-ray diffractometer (Cu Kα radiation source). The measurement was performed on a ZnO wire array grown on a silicon nitride (SiN, 100 nm)/Si bulk substrate using the same process conditions as for the SiN nanomembrane (NM). Note that the X-ray beam illuminated the whole ZnO wire area, thus, the measurement is an average over all wires.

The diffraction pattern is displayed in Figure S2. XRD peaks from hexagonal ZnO crystal structure are clearly present as indexed in the figure (reference database: [2]). In comparison to ZnO powder XRD measurements [3], where the (101)-peak is the most intense peak, the strong (002)-peak in Figure S2 indicates a preferred ZnO wire crystal growth along the (002) direction.

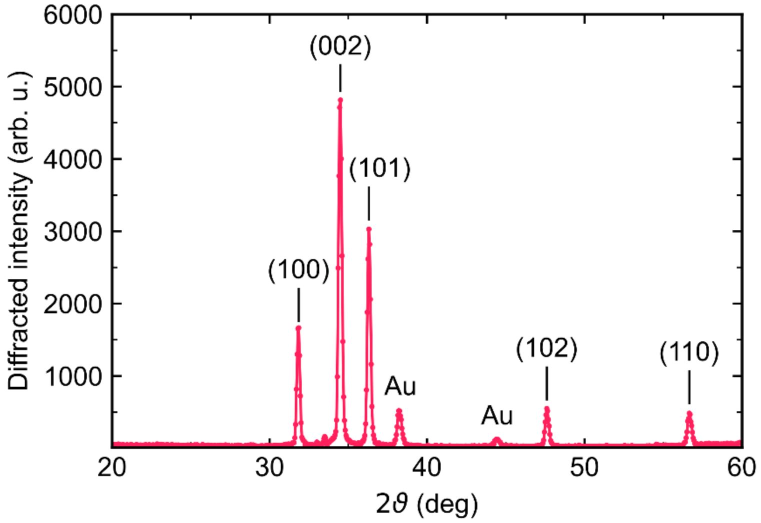

Figure S2. X-ray diffraction measurement on ZnO wires on a silicon bulk substrate. The peak indexing corresponds to the hexagonal ZnO crystal structure [2]. The Au peaks refer to the fcc-Au crystal structure.

Overlay of the measured mass spectra

Figure S3 displays the 100 mass spectra that were measured for each protein concentration. To allow for the analysis of the peak abundance in dependence on the *m/z* value despite the considerable background signal, the data is presented in the form of histograms in Figure 2.

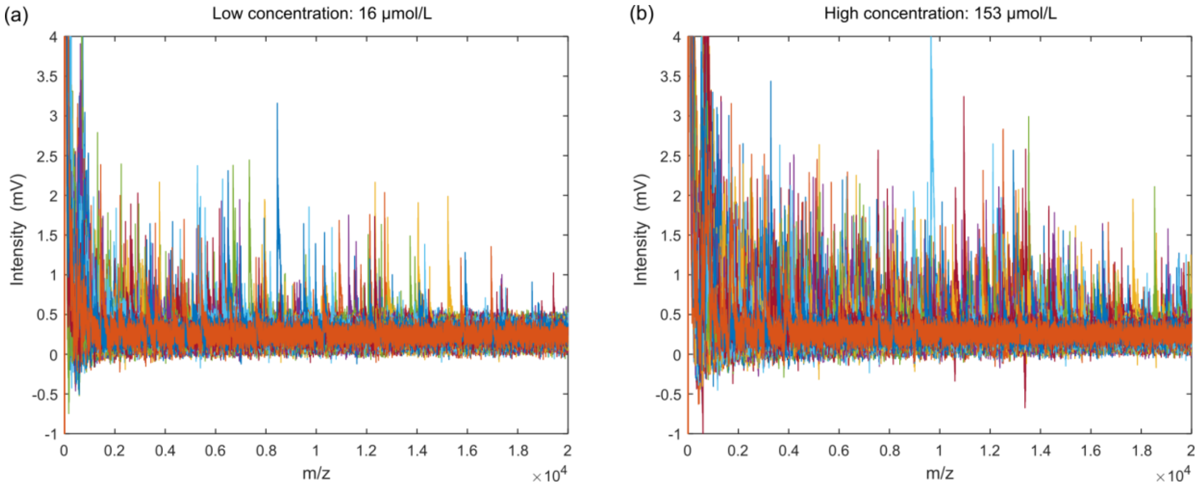

Figure S3. Overlay of the 100 mass spectra that were measured for (a) low and (b) high protein concentration.

Zoomed view of the singly charged ion peak

Figure S4 shows the increase in count number for the *m/z* value of about 12,384 (gray vertical line), which was attributed to the detection of the singly charged cytochrome c ion. A Gaussian fit (green line) was used to extract the mean count number of 18.4 ± 0.5 for the high protein concentration (153 µmol/L). A mean count number of 4.5 ± 0.2 was found for the low protein concentration (16 µmol/L).

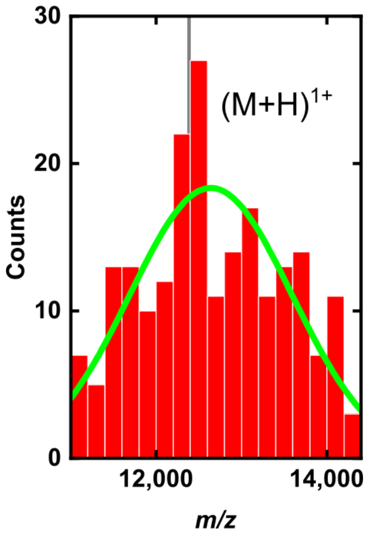

Figure S4. Zoomed view of the peak that appeared in the histogram for the singly charged cytochrome c ion at about *m/z* 12,384 (high protein concentration) with a Gaussian fit (green line).

Peak intensity versus protein concentration

The prominence of the peaks that appeared for the singly charged cytochrome c ion were directly extracted from the mass spectra for both protein concentrations.

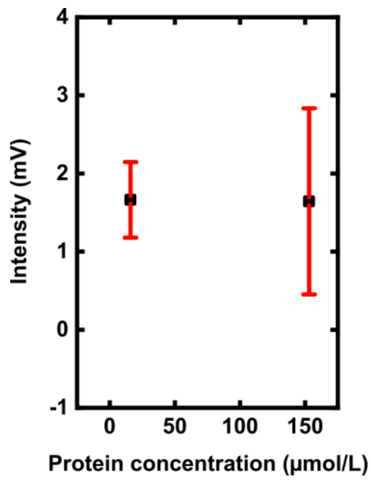

Figure S5. Mean peak intensity for different protein concentrations.

Variation of Fermi level with doping and temperature

Field emission (FE) is closely related to the work function of the emitting material, which, in a semiconductor, depends on the sum of its electron affinity and the difference between the conduction band edge and the Fermi level $E_C - E_F$. That difference is related to doping via carrier concentration $E_C - E_F = k_B T ln\left(\frac{N_C}{n}\right)$, where k_B is the Boltzmann constant and N_C is the effective density of states in the conduction band. Temperature has a large impact on this separation and at high temperatures, the material will become nearly intrinsic as the Fermi level returns to the intrinsic level. In order to understand the impact of doping on the FE current, we calculated the position of the Fermi level as a function of temperature in our simulations, shown in Figure S6, where we see that heating causes the Fermi level to approach the intrinsic level regardless of doping. From Figure S7, we observe that, as the temperature increases, the Fermi level of the device reduces to its intrinsic value, from which we conclude that doping the NM to move the Fermi level would not significantly impact performance at high temperatures at which most of the FE occurs here.

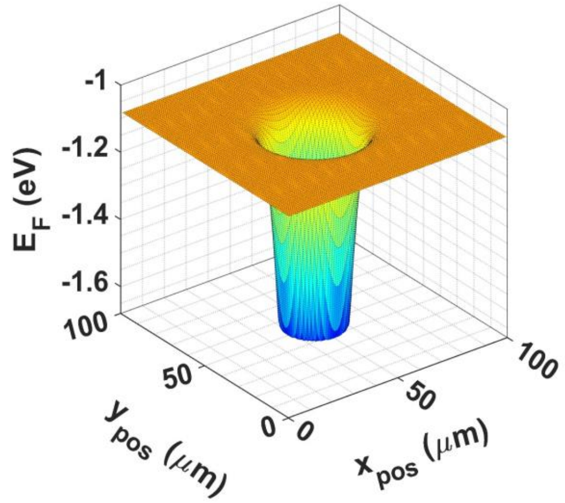

Figure S6. The Fermi level (E_F) profile as a function of position across the NM showing that E_F decreases with temperature and is lowest at the center of the NM, saturating at its intrinsic value where the temperature is highest.

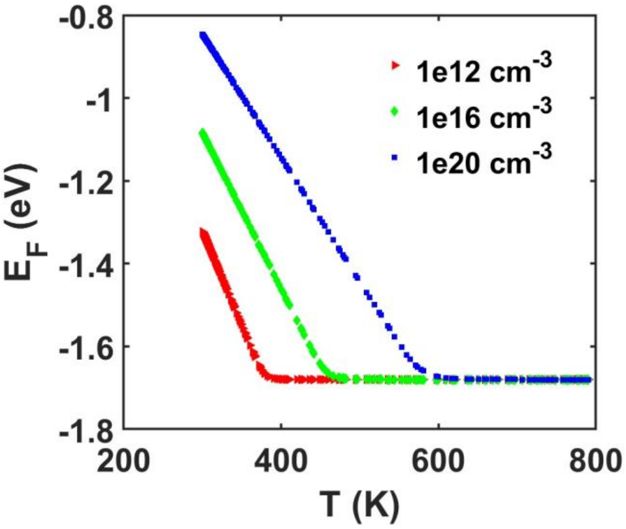

Figure S7. E_F plotted as a function of temperature for various doping concentrations showing that, at high temperatures, the Fermi level returns to the intrinsic level mid-gap.

Impact of wire length and diameter variation

The lengths of the wires in our simulation were sampled from a distribution plotted in Figure S8 (a) and used to calculate the FE current as a function of time (b). We varied the width of the distribution while keeping the mean constant at 50 micrometers and found that, for symmetric distributions, the total current remains very close to the current from uniform wires having the same length as the mean of the distribution. Similarly, we varied the diameter of the wires in Figure S8 (c) around the mean of 2.3 micrometers. The resulting current stays very close to the one obtained having uniform diameter equal to the mean of the distribution, regardless of the variance of width.

However, when we explored the impact of the skew of the distributions of wire widths and lengths, we saw some change. The distributions of lengths and widths are shown in Figure S9 (a) and (c), respectively. When the distribution is heavily skewed toward larger or smaller lengths or diameters, then the current deviates from the current obtained from uniform wires, as shown in Figure S9 (b) for length and (d) for diameter variation. As we see from Figure S1, the distributions of lengths and diameters are fairly broad but do not show significant skew.

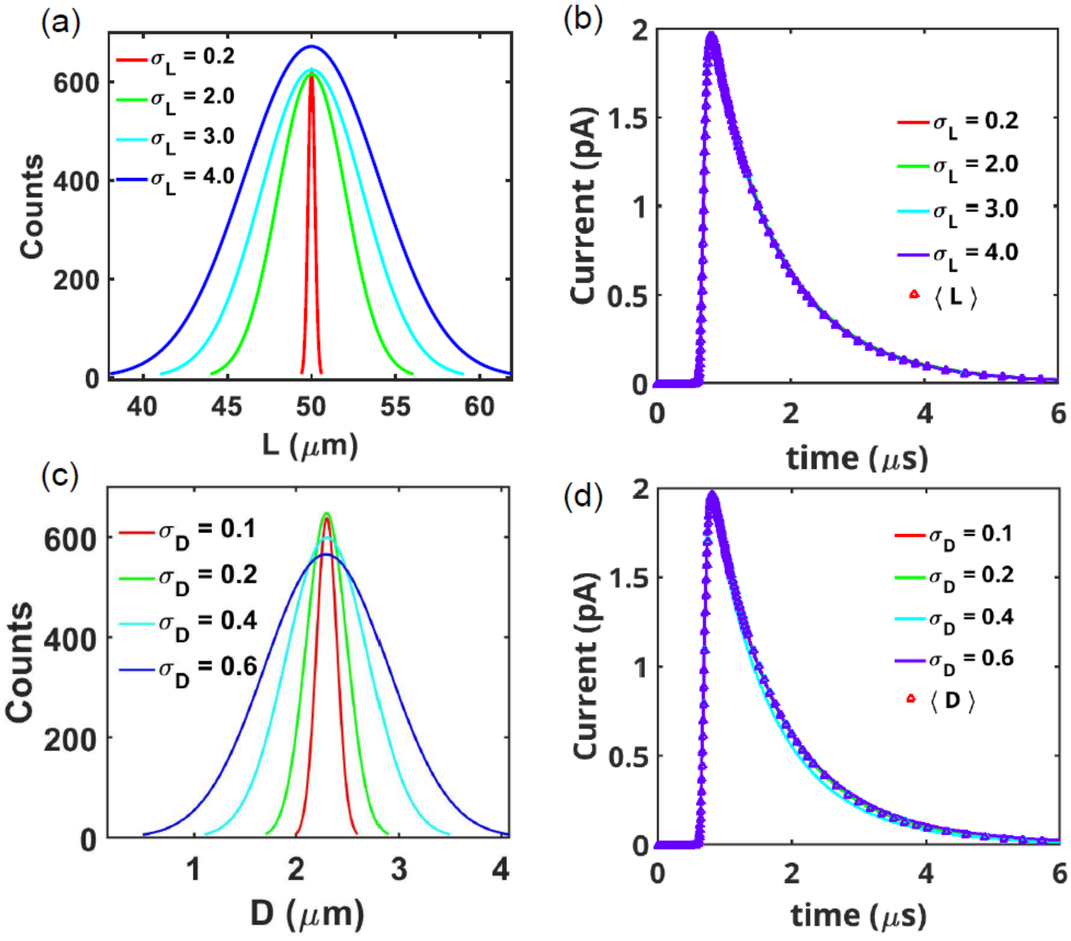

Figure S8. (a) Gaussian distribution of wire lengths with different standard deviations and uniform mean value of 50 μm. (b) FE current from wires with length distributions shown in 'a' and uniform wire diameters of 2.3 μm. The FE current is the same for all distributions and is equal to that calculated using wires with lengths that are equal to the mean of the distributions. (c) Gaussian distribution of wire diameters with different standard deviations and uniform mean value of 2.3 μm. (d) FE current from the wires with diameter distributions shown in 'c' for uniform wire lengths of 50 μm. The symbols show FE current when all the wires have a diameter equal to the mean of the corresponding distribution. We observe that the FE current is the same and equal to the FE current at the mean value of the distribution.

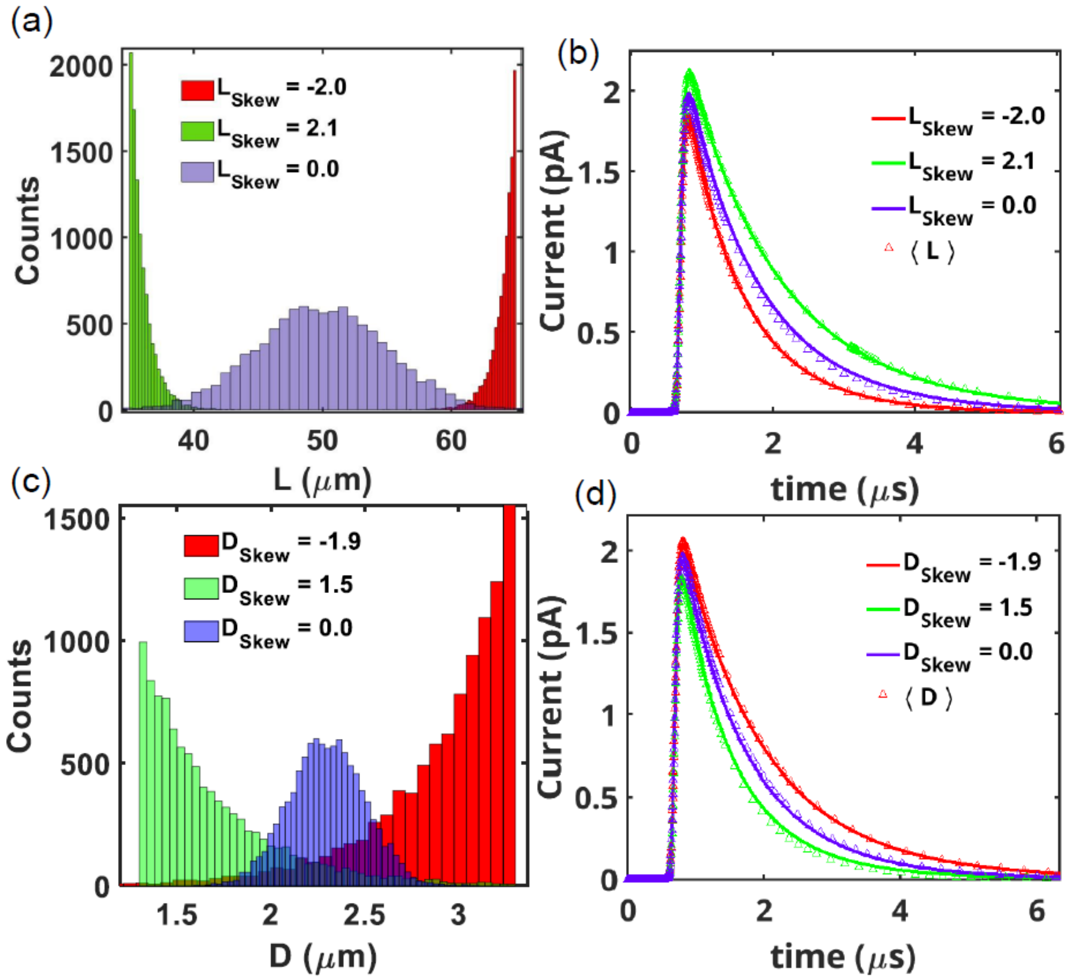

Figure S9. (a) Distributions of wire lengths with varying skewness and mean of 36 µm, 49 µm, and 64 µm. (b) FE current from wire length-distributions shown in 'a' and uniform wire diameter of 2.3 µm. The FE current is larger for length-distributions with a positive skewness, but in all cases the FE current from wires of randomly distributed lengths is the same as that from wires with lengths equal to the mean value shown using symbols. (c) Distributions of wire diameter with different skewness and mean values of 1.7 µm, 2.3 µm, and 2.9 µm. (d) The FE current from wires with randomly distributed diameters is the same as that calculated using the mean value of the diameters which is shown using symbols. The FE current is larger for a diameter distribution that is negatively skewed.

Rise and fall times of the singly charged ion peak

The rise and fall times for the peak that appeared for the singly charged cytochrome c ion at about *m/z* 12,384 were extracted as the time between the maximum of the peak and 10% of its total intensity.

Table S1. Summary of rise and fall times for low and high protein concentrations.

Protein concentration / µmol/L	Mean *m/z*	Rise time / µs	Fall time / µs
16	12,373 ± 216	0.02 ± 0.03	1.9 ± 1.2
153	12,383 ± 175	0.04 ± 0.14	1.3 ± 1.2

Mass resolution versus *m/z* value

The mass resolution (m/Δm) given in previous publications from measurements with pristine nanomembranes as detectors are summarized below [4], [5]. The values of m/Δm are shown for a Si membrane (plotted in black), a SiN membrane covered with aluminum from both sides (green), and for the herein investigated, surface-modified nanomembrane detector (red).

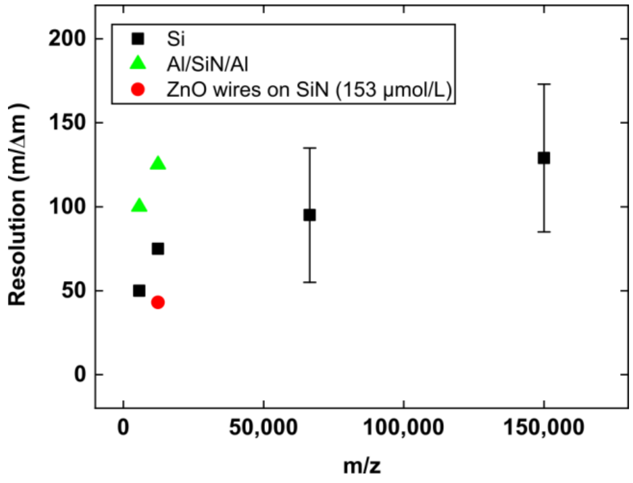

Figure S10. Mass resolution (m/Δm) as a function of the *m/z* value. The values for the Si and for the Al/SiN/Al membrane were taken from previous publications [4], [5].

Example for rise and fall time

The rise and fall time were extracted from the measurement of the signal intensity in dependence on the flight time for peaks that appeared in the range of 112.3 µs to 115.9 µs, which corresponds to about *m/z* 12,384 ± 400. Ten spectra were found for the low protein concentration and 47 spectra for the high protein concentration with a peak within this flight time range, respectively. An example of the time-dependent behavior of the cytochrome c peak is shown in Figure S11. Note, the experimental data used for rise and fall time extraction was smoothed with the MATLAB function “mslowess” (MathWorks).

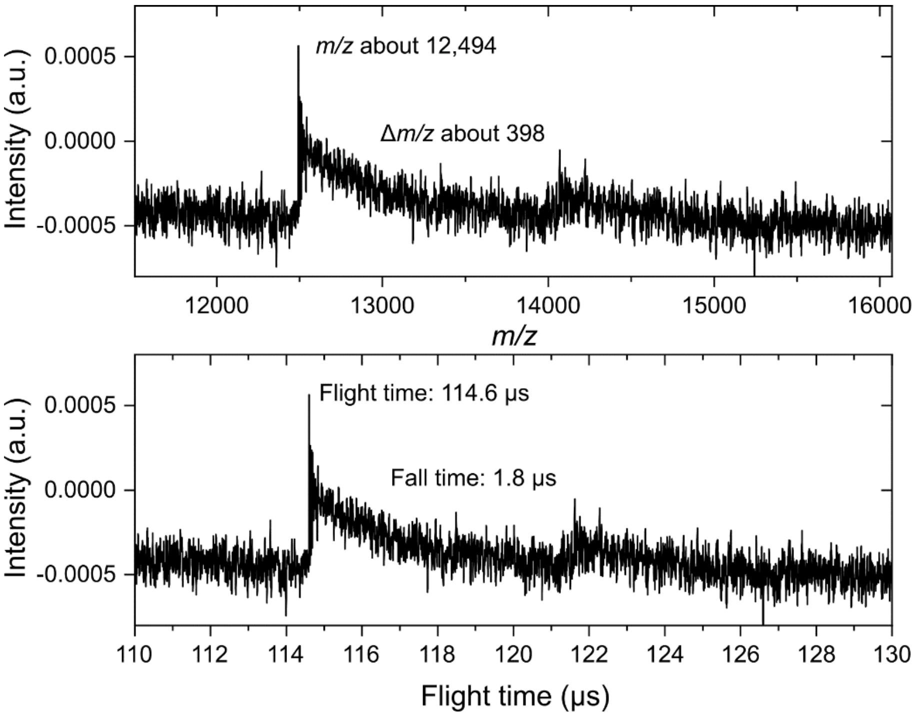

Figure S11. The experimental data is shown in dependence on the calculated *m/z* values (top) and the measured flight time (bottom). The peak that appeared for the singly charged cytochrome c around an expected flight time of about 114 µs, shows a steep rise time, followed by an exponential decrease within around 1.8 µs.

Measurement with conventional MCP detector

The rise and fall times were extracted from a MALDI-TOF measurement using a conventional MCP detector without a nanomembrane.

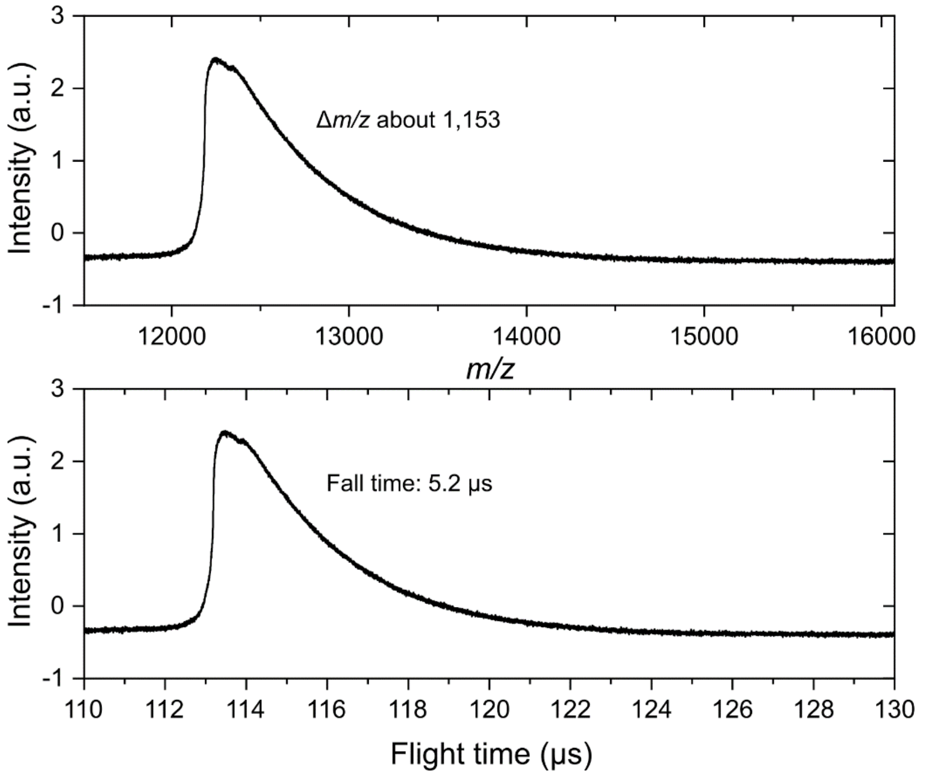

Figure S12. The experimental data measured with a conventional MCP detector is shown in dependence on the calculated *m/z* values (top) and the measured flight time (bottom).

SEM images of particularly large ZnO wires

A mean aspect ratio of 21.7 was determined for the herein investigated type of ZnO wires (50 µm length and 2.3 µm diameter). Note, several ZnO wires can be found with considerably larger aspect ratios on a sample that was fabricated using the same growth parameters. The two examples in Figure S13 have an aspect ratio of about 64 (wire #1: 83 µm length, 1.3 µm diameter) and 31 (wire #2: 68 µm length, 2.2 µm diameter). Note, the wire dimensions were measured using ImageJ [1] and the vertical length measurements were corrected for the SEM stage tilt angle of 54° [6].

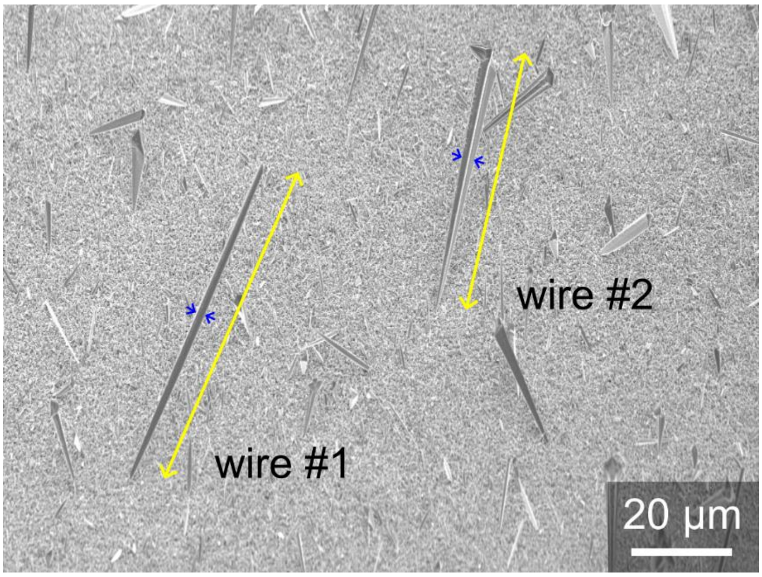

Figure S13. Outstandingly large ZnO wires on a free-standing SiN NM (100 nm thickness, 5x5 mm² membrane size). The length and diameter measurements are indicated by yellow and blue lines, respectively.

References

[1] Schneider, C. A.; Rasband, W. S.; Eliceiri, K. W. NIH Image to ImageJ: 25 Years of Image Analysis. *Nat. Methods* **2012**, 9, 671–675. https://doi.org/10.1038/nmeth.2089.

[2] Reference database: Inorganic Crystal Structure Database; Coll. Code: 44477

[3] Šarić, A.; Vrankić, M.; Lützenkirchen-Hecht, D.; Despotović, I.; Petrović, Ž.; Dražić, G.; Eckelt, F. Insight into the Growth Mechanism and Photocatalytic Behavior of Tubular Hierarchical ZnO Structures: An Integrated Experimental and Theoretical Approach. *Inorg. Chem.* **2022**, 61 (6), 2962–2979. https://doi.org/10.1021/acs.inorgchem.1c03905.

[4] Park, J.; Aksamija, Z.; Shin, H.-C.; Kim, H.; Blick, R. H. Phonon-Assisted Field Emission in Silicon Nanomembranes for Time-of-Flight Mass Spectrometry of Proteins. *Nano Lett.* **2013**, 13 (6), 2698–2703. https://doi.org/10.1021/nl400873m.

[5] Kim, H.; Park, J.; Aksamija, Z.; Arbulu, M.; Blick, R. H. Ultrananocrystalline Diamond Membranes for Detection of High-Mass Proteins. *Phys. Rev. Appl.* **2016**, 6 (6), 064031. https://doi.org/10.1103/PhysRevApplied.6.064031.

[6] Goldstein, J. I.; Newbury, D. E.; Joy D. C.; Lyman, C. E.; Echlin, P.; Lifshin, E.; Sawyer, L.; Michael, J. R. Image Formation and Interpretation. In *Scanning Electron Microscopy and X-ray Microanalysis*, 3rd ed.; Springer, Boston, MA, 2003; pp. 119–123.
